# Associations between lipid-lowering drugs and urate and gout outcomes: a Mendelian randomization study

**DOI:** 10.3389/fendo.2024.1398023

**Published:** 2025-01-24

**Authors:** Min Liu, Na Yin, Yuhang Zhu, Aili Du, Chunyuan Cai, Pengyuan Leng

**Affiliations:** ^1^ Department of Orthopaedics, The Third Affiliated Hospital of Wenzhou Medical University, Wenzhou, China; ^2^ College of Nursing, Hangzhou Normal University, Hangzhou, China

**Keywords:** drug targets, eQTL, gout, lipid-lowering drugs, Mendelian randomization, urate

## Abstract

**Objective:**

Gout is a common inflammatory arthritis and lipid metabolism plays a crucial role in urate and gout. Potential associations between urate and gout and lipid-lowering drugs have been revealed in observational studies. However, the effects of lipid-lowering drugs on urate and gout remain controversial. The aim of this study was to investigate the genetic association between lipid-lowering drugs and urate and gout.

**Methods:**

In this study, two genetic proxies were employed to approximate lipid-lowering drug exposure: expression quantitative trait loci (eQTL) associated with drug-target genes and genetic variations proximal to or within genes targeted by these drugs, which are linked to low-density lipoprotein cholesterol (LDL-C) levels. The study’s exposures encompassed genetic variants within drug target genes (HMGCR, PCSK9, NPC1L1), each representing distinct lipid-lowering mechanisms. Causal effects were estimated using the inverse variance weighting (IVW) method, while the Summary Data-based Mendelian Randomization (SMR) method, leveraging pooled data, was applied to compute effect estimates. These estimates were further refined through various approaches including MR-Egger, the weighted median method, simple and weighted models, and leave-one-out analyses to conduct sensitivity analyses.

**Result:**

The analytical outcomes utilizing the IVW method indicated that inhibitors of HMGCR were correlated with an elevated risk of developing gout (IVW: OR [95%CI] = 1.25 [1.03, 1.46], p=0.0436), while PCSK9 inhibitors were linked to heightened levels of urate (IVW: OR [95%CI] = 1.06 [1.01,1.10], p=0.0167). Conversely, no significant correlation between NPC1L1 inhibitors and the levels of urate or the risk of gout was established. Furthermore, the SMR analysis failed to identify significant associations between the expression levels of the HMGCR, PCSK9, and NPC1L1 genes and the risk of gout or elevated urate levels (SMR method: all P values >0.05). Sensitivity analyses further confirmed the robustness of these results, with no significant heterogeneity or pleiotropy found.

**Conclusion:**

This study furnishes novel causal evidence supporting the potential genetic correlation between the use of lipid-lowering drugs and the incidence of gout as well as urate levels. The findings indicate that inhibitors targeting HMGCR may elevate the risk associated with the development of gout, while inhibitors targeting PCSK9 are likely to increase urate concentrations.

## 1 Introduction

Urate, the terminal product of purine metabolism, manifests an imbalance between its synthesis and excretion, potentially leading to increased serum urate concentrations and the genesis of monosodium urate crystals (1). Gout represents an inflammatory condition, instigated by the deposition of monosodium urate crystals within joints and soft tissues, and stands as the globally most widespread variant of inflammatory arthritis (2).This condition places a significant burden on individual health and the healthcare system (3). Gout afflicts approximately 4% of the adult population in developed nations, with over 7 million new cases emerging globally each year (4), and its prevalence is notably increasing alongside economic development (5). The disease is typified by recurrent acute episodes, which may culminate in severe pain and significant dysfunction (6). Severe gout can lead to chronic kidney diseases or urinary tract stones, potentially resulting in kidney failure (7). Studies show that dyslipidemia increases the risk of gout and higher serum urate levels (8). Dyslipidemia involves high levels of total cholesterol, triacylglycerols, and low-density lipoprotein cholesterol (LDL-C), which are all positively linked to serum urate levels (9). Dyslipidemia occurs more frequently in gout patients than in those with silent high urate levels (10) and gout patients often have a history of dyslipidemia (11).

Statins are preferred for the treatment of dyslipidemia due to their proven efficacy in reducing LDL-C levels and mitigating the risk of atherosclerotic cardiovascular disease (ASCVD) (12). Inhibitors of 3-Hydroxy-3-methylglutaryl coenzyme A reductase (HMGCR), including simvastatin and rosuvastatin, represent prevalent statin types. Additionally, other lipid-lowering agents, including FDA-approved inhibitors of the preprotein convertase subtilisin/kexin type 9 (PCSK9) and ezetimibe targeting Niemann-Pick C1-like 1 (NPC1L1), are employed to augment the lipid-reducing efficacy (13, 14). PCSK9, identified as a serine protease, occupies a pivotal role in the regulation of LDL-C metabolism, contributing to the onset of dyslipidemia and atherosclerosis through the inhibition of LDLR recycling to the cellular surface, thus elevating LDL-C concentrations (15). NPC1L1, a transmembrane protein prevalent in diverse cells, notably within the parietal membrane of intestinal epithelial cells and the renal tubular membrane of hepatocytes, plays a crucial role in mediating cholesterol absorption and overseeing hepatic cholesterol excretion (16), significantly influencing LDL-C metabolism regulation (17). Extant research highlights that both statin and non-statin lipid-lowering medications may contribute to increased urate levels and a heightened risk of gout development (12, 18), While certain investigations have probed into the correlation between serum urate and lipid concentrations, the findings remain contentious (19). Therefore, it has become particularly important to thoroughly investigate the causal relationship between lipid-lowering drugs and urate levels and gout.

Drug target Mendelian randomization analyses utilize genetic variation that mimics the pharmacological inhibitory effects of a pharmacogenetic target as an instrumental variable (IV). This approach aims to clarify the consequences of drug utilization via regression techniques, thereby augmenting the comprehension of the causal nexus between drug targets and the potential repercussions on urate levels and gout manifestations (15). In accordance with Mendel’s laws, genetic material undergoes random distribution during meiosis and is transmitted from parents to offspring during fertilization, thereby reducing the likelihood that the outcomes of MR studies are influenced by potential confounding factors or reverse causation (20). Consequently, MR analyses have furnished a tier of evidence that is second only to that provided by randomized controlled trials (21). In the current investigation, we employed a two-sample Mendelian Randomization analysis approach to explore the relationship between lipid-lowering agents (HMGCR inhibitors, PCSK9 inhibitors, and NPC1L1 inhibitors) and outcomes related to urate and gout.

## 2 Materials and methods

### 2.1 Study design

**Figure 1:**
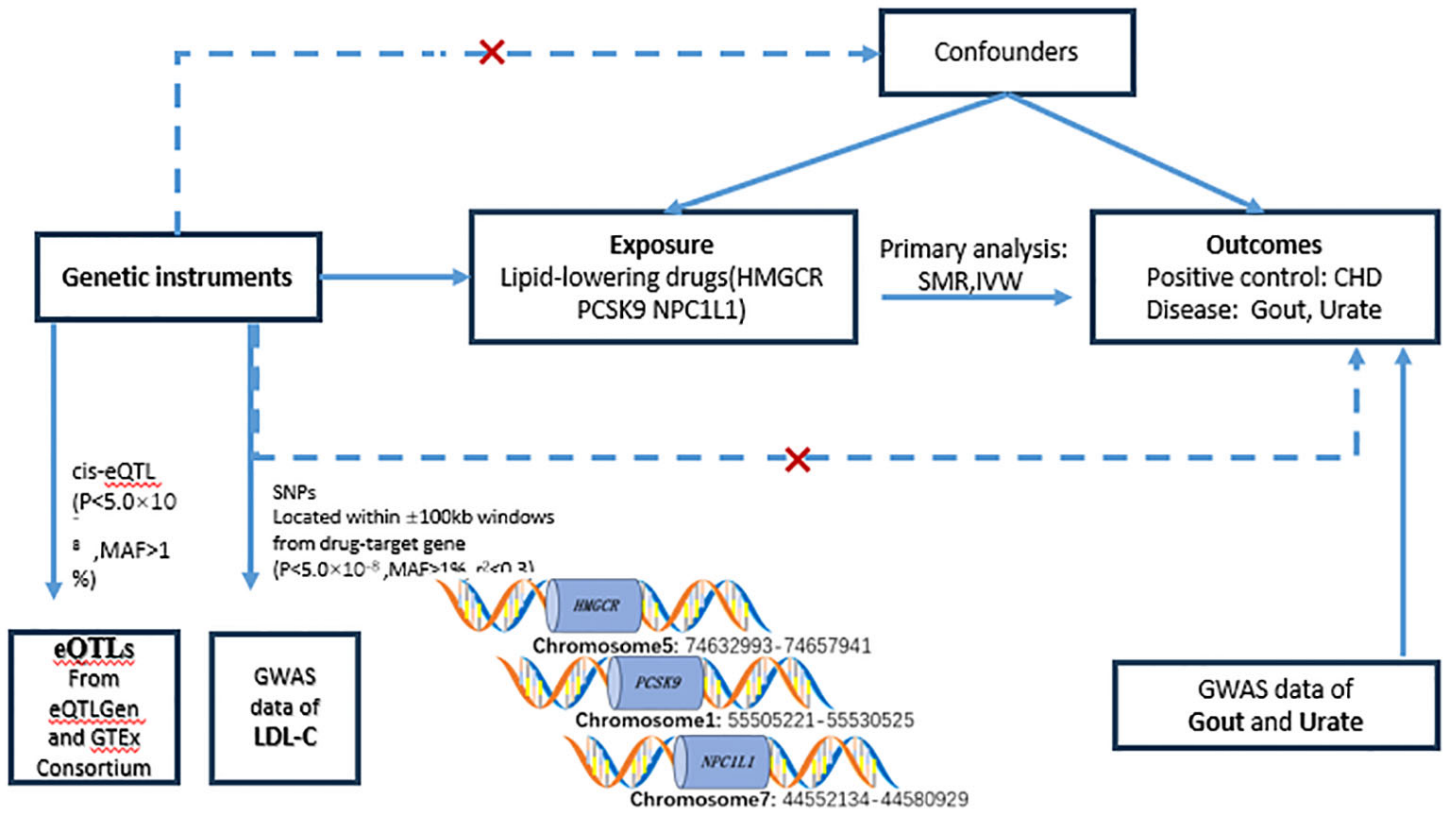
Flowchart of the study design. In order to study causality, the following conditions need to be met: (1) genetic variation should be strongly correlated with exposure (solid line); (2) genetic variation should not be correlated with confounders (dashed line); and (3) genetic variation should not have a direct relationship with the outcome (dotted line). eQTL, expressed quantitative trait loci; SNP, single nucleotide polymorphism; MAF, minor allele frequency; SMR, Mendelian randomization based on pooled data; IVW-MR, inverse variance weighted Mendelian randomization; LDL, HMGCR, 3-hydroxy-3- methylglutaryl-coenzyme A reductase; PCSK9, preprotein convertase Bacillus subtilis protease/kexin type 9; NPC1L1, Niemann-Pick C1-like 1; CHD, coronary heart disease; Gout, gout; Urate, uric acid.

This investigation adhered to the guidelines stipulated by the STERBE-MR framework (22), Additionally, a two-sample Mendelian Randomization analysis was employed to evaluate the influence of drug targets. The analytical framework was grounded on publicly accessible summary-level data derived from Genome-Wide Association Studies (GWAS) and Expression Quantitative Trait Loci (eQTL) investigations. Two principal methodologies were utilized in the analysis: Summary Data-based Mendelian Randomization (SMR) and Inverse Variance Weighted Mendelian Randomization (IVW-MR) techniques. Detailed information and specific data sources are delineated in the annex (**Supplementary Table S1**)with the study’s flowchart presented in **Figure 1**. The foundational GWAS study underpinning this research received approval from pertinent ethical review boards, with participants providing signed informed consent forms. Given that only summary-level data from publicly accessible genetic databases were utilized, no further ethical approvals were necessitated for this study (23).

### 2.2 Selection of genetic instruments

In this investigation, we identified genes encoding the target proteins of currently available LDL-lowering drugs using the DrugBank database, three categories of lipid-lowering agents were designated as exposure variables: HMGCR, PCSK9, and NPC1L1 inhibitors. Serving as surrogates for these drug exposures, accessible eQTL datasets were utilized, encompassing only those eQTLs expressed in serum, liver, or adipose tissues associated with lipid-lowering target genes, which were identified as common cis-eQTLs. A significance threshold was established at P < 5.0 × 10^-8, with a minor allele frequency (MAF) exceeding 0.01. During the data analysis phase, the HMGCR, PCSK9, and NPC1L1 genes presented 921, 24, and 11 cis-eQTLs, respectively, aligning with their corresponding drug targets. For every targeted gene, the most representative cis-eQTL SNPs were chosen as genetic instruments for the analysis. Data from the eQTL Gen consortium (24) (https://www.eqtlgen.org/) or GTEx Alliance V8 (25) (https://gtexportal.org/). Furthermore, we evaluated the linkage between individual genetic variants and levels of LDL-C. Aggregated data concerning LDL-C levels were sourced from the Global Lipids Genetics Consortium (GLGC) (23), encompassing 173,082 individuals of European descent. Single nucleotide polymorphisms (SNPs) were utilized as surrogate markers for exposure to LDL-C lipid-lowering drugs. Genetic instruments based on SNPs were chosen according to specific criteria: they were situated within or in close proximity to the ±100 kb region surrounding the pertinent drug target gene and exhibited a high correlation with it (p<5.0×10^-8). In order to augment the robustness of the instrumental variables, SNPs were permitted to exhibit low weak linkage disequilibrium (r^2^< 0.30). Ultimately, utilizing data from the GLGC (ieu-a-300), we identified and selected 7, 12, and 3 significant SNPs within the HMGCR, PCSK9, and NPC1L1 genes, respectively, as our genetic instruments (**Supplementary Table S2**).

### 2.3 Sources of results

In this investigation, urate and gout data were employed as the principal outcome measures for the execution of Mendelian Randomization analyses concerning drug targets. To ascertain the robustness of our results, coronary heart disease (CHD) was incorporated as a positive control, reflecting the well-documented efficacy of lipid-lowering therapies in diminishing CHD incidence rates (26, 27). The CHD dataset was sourced from GWAS summary statistics, encompassing 60,801 cases and 123,504 controls (28). For the condition of gout, summary statistics were derived from a cross-ethnic meta-analysis of GWAS, which involved 763,813 participants and included 13,179 gout cases. Conversely, GWAS data pertaining to urate were procured from a cross-racial meta-analysis incorporating 457,690 individuals across 74 studies (29)(**Supplementary Table S1**).

### 2.4 Statistical analysis

#### 2.4.1 Primary MR analysis

In the present investigation, we employed a SMR approach, utilizing eQTL as a tool to harness pooled data from GWAS and eQTL studies for probing the relationship between gene expression levels and their correlation outcomes. Furthermore, to ensure the stability of the observed associations, HEIDI tests were conducted. All analyses were executed utilizing the SMR software version 1.03. (for details see:(https://cnsgenomics.com/software/smr/#Overview). When employing SNPs as genetic instruments, alongside the primary application of IVW-MR methods, additional analyses, including weighted median, simple modal, weighted modal, and MR-Egger regression, were undertaken to augment the comprehensiveness and depth of the investigations. The results of these analyses are presented in **Supplementary Table S3**.

#### 2.4.2 Sensitivity analysis

In order to ascertain the robustness of the instrumental variables (IVs), we evaluated their strength employing the F-statistic as a metric. Exclusively, single nucleotide polymorphisms (SNPs) exhibiting an F-statistic exceeding 10 were incorporated to mitigate the bias originating from insubstantial instruments (30). Moreover, statistical efficacy was assessed via the mRnd website(https://shiny.cnsgenomics.com/mRnd/). Within the framework of the SMR approach, the link between gene expression and outcome variables was evaluated by conducting a HEIDI test to determine if the observed association stems from linkage disequilibrium (31), A p-value lower than 0.01 indicates a potential for linkage disequilibrium. To enhance the robustness assessment of the Mendelian Randomization outcomes, we employed the intercept test and the Cochran Q test within the MR Egger regression framework to evaluate the potential degrees of multicollinearity and heterogeneity (32, 33). The MR Egger regression and MR-PRESSO methods were employed to evaluate the horizontal pleiotropy of genetic instruments. A p-value greater than 0.05 indicates the absence of horizontal pleiotropy (34). Conversely, Cochran’s Q test is employed to assess heterogeneity, where a p-value below 0.05 signifies substantial heterogeneity (35). Furthermore, a leave-one-out analysis was conducted to ensure the reliability of the overall effect (36). This analysis was conducted as part of the sensitivity analyses, where each instrumental SNP was sequentially excluded to evaluate the causal impact of the remaining SNPs on the outcome, thereby determining if the MR findings were influenced by any specific SNP. Ultimately, the credibility of the genetic instrument was corroborated through the execution of a positive control study. The analysis utilized R version 4.3.1, employing the TwoSampleMR and MR-PRESSO packages (36, 37).

## 3 Results

### 3.1 Preliminary analysis

**Figure 2:**
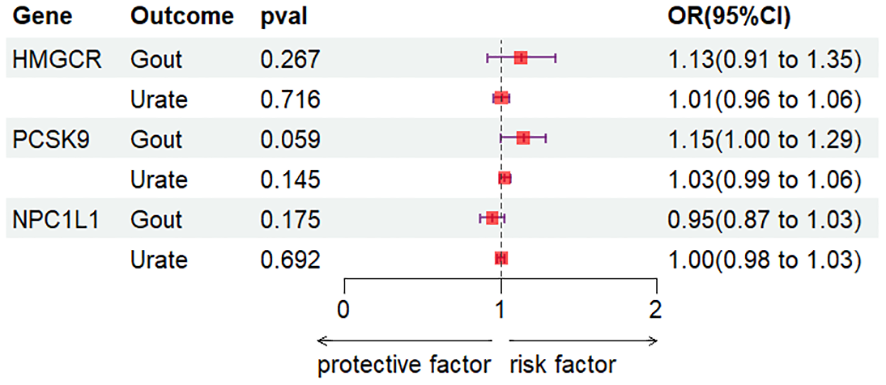
SMR analysis.

In this investigation, the correlation between gene expression of HMGCR, PCSK9, and NPC1L1 and outcomes related to gout and urate was evaluated utilizing the SMR approach. Cis-eQTL results for HMGCR, PCSK9, and NPC1L1 were sourced from the eQTLGen and GTEx consortia, featuring 921, 24, and 11 SNPs, respectively. Following this, the most indicative cis-eQTL SNPs (rs6453133, rs472495, and rs41279633 for each gene respectively) were chosen as instrumental variables (IV) for their corresponding drug target genes and subjected to analysis via the SMR method. The results showed no significant association between elevated levels of HMGCR, PCSK9, and NPC1L1 gene expression and the risk of gout and uric acid (all p-values > 0.05) (**Figure 2**). Additionally, the outcomes of the HEIDI test indicated that the observed correlations were not influenced by genetic linkage (all p-values > 0.05).

**Figure 3:**
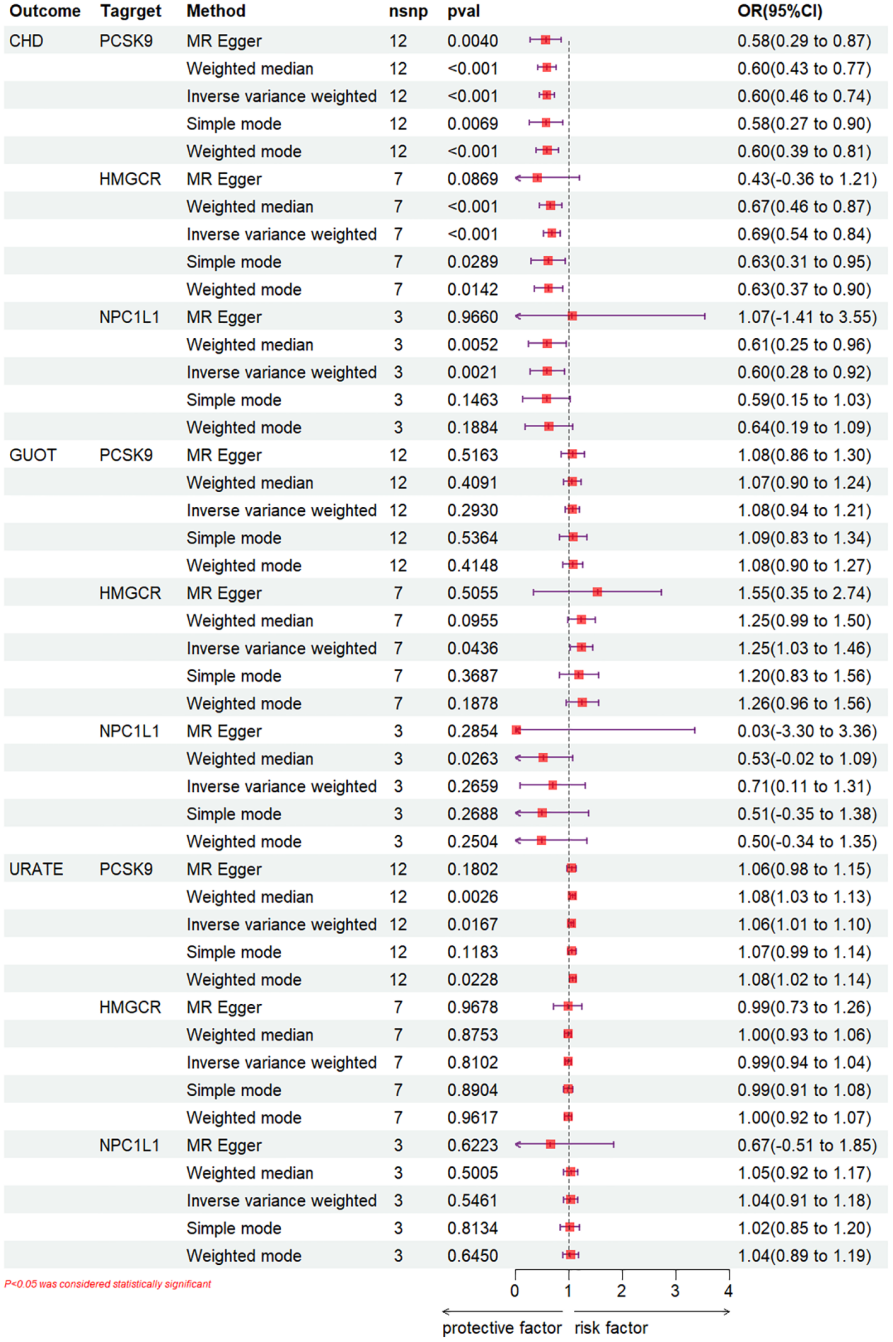
IVW-MR analysis.

IVW-MR analysis demonstrated (**Figure 3**) a correlation between elevated expression of the HMGCR gene and a heightened risk of gout (OR [95%CI] = 1.25 [1.03, 1.46], p = 0.0436). Weighted median analysis indicated that NPC1L1 inhibitors could potentially act as a risk factor for gout (OR [95%CI] = 0.53 [-0.02, 1.09], p = 0.0263). Concurrently, IVW analysis along with weighted median and weighted mode analyses uniformly revealed that PCSK9 inhibitors may elevate the risk of urate (IVW: OR [95%CI] = 1.06 [1.01, 1.10], p = 0.0167; weighted median: OR [95%CI] = 1.08 [1.03, 1.13], p = 0.0026; weighted mode: OR [95%CI] = 1.08 [1.02, 1.14], p = 0.0228). Regarding NPC1L1 expression, no significant correlation with gout and urate levels was established (**Supplementary Files S1**-**S4**).

### 3.2 Positive control analysis

To ascertain the precision of the study’s outcomes, a positive control analysis was conducted. An examination of the GLGC dataset employing the IVW method revealed that PCSK9, HMGCR, and NPC1L1 inhibitors significantly mitigated the risk of CHD (OR [95%CI] =0.60 [0.46-0.74], p<0.001; OR [95%CI] =0.69 [0.54-0.84], p<0.001, respectively) (OR [95%CI] =0.60 [0.28-0.92], p=2.1×10^-3) (**Figure 3**), (**Supplementary Files S1**-**S5**).

### 3.3 Sensitivity analysis

For the evaluation of heterogeneity and horizontal pleiotropy, the Cochrane Q-test and MR Egger regression models were employed (33). In the IVW-MR analyses, neither significant heterogeneity nor horizontal pleiotropy was observed, with all p-values exceeding 0.05, indicating the robustness of the findings. (**Supplementary Files S1**-**S6**). The MR-Egger regression and MR-PRESSO analyses provided no evidence of potential horizontal pleiotropy. Our comprehensive leave-one-out sensitivity analyses revealed that the exclusion of any single SNP did not lead to significant alterations in the effect estimates. This further reinforces the robustness of our findings, indicating that they are independent of the influence exerted by any individual SNP (**Supplementary File 2**). Through the execution of these sensitivity analyses, the reliability of the findings was affirmed and the impact of potential confounders was minimized.

## 4 Discussion

In this investigation, we employed a comprehensive approach to explore the impact of lipid-lowering drug targets on gout and urate through drug-target Mendelian Randomization and SMR analysis. Utilizing genetic instruments, our goal was to surmount the constraints inherent in observational studies and to furnish more robust evidence for the potential role of HMGCR, PCSK9, and NPC1L1 in the onset of gout and urate. The findings from our analysis indicated no significant associations between SMR-based gene expression levels of HMGCR, PCSK9, and NPC1L1 and the risks of developing gout and elevated urate levels. However, through IVW-MR analysis, we identified a positive relationship between HMGCR inhibition and gout, albeit without a significant correlation to hyperuricemia. Conversely, a positive relationship was observed between PCSK9 inhibition and hyperuricemia, while no genetic association with gout was detected. The analysis indicated no significant causal link between the gene expression of NPC1L1 inhibitors and the onset of gout and urate levels, implying that the side effects associated with NPC1L1 inhibitors in patients suffering from gout and urate might be inferior to those arising from HMGCR and PCSK9 inhibitors. These results offer theoretical backing for tailoring hyperlipidemia treatment approaches in individuals with gout and hyperuricemia. The rising concern of pharmacologically induced hyperuricemia and gout in clinical settings is noteworthy. Various medications, particularly diuretics, antituberculosis agents, and immunosuppressants, have been implicated in triggering hyperuricemia accompanied by gout (38). Observational research has indicated that instances of hyperuricemia and gout are prevalent among individuals suffering from dyslipidemia (39–41), A comprehensive randomized, double-blind, placebo-controlled trial involving 13,970 participants revealed that individuals on lipid-lowering medication exhibited an increased risk of developing gout and hyperuricemia in comparison to those in the placebo group (42). Nevertheless, current investigations into its underlying mechanisms remain inadequate, and the conclusions drawn from various studies are inconsistent. For instance, a retrospective analysis discovered that patients administering a combination of allopurinol, febuxostat, and fenofibrate experienced a more significant reduction in serum urate concentrations (43). Conversely, another observational study highlighted that fenofibrate frequently correlates with nephrotoxicity among gout patients, underscoring the necessity for additional investigations into the selection of lipid-lowering medications in the treatment of gout (44). The reliability of these findings is controversial due to the scarcity of randomized controlled trials and limited cohort studies. A meta-analysis of Phase 2 and Phase 3 clinical studies shows that bempedoic acid raises the risk of hyperuricemia and gout (45). In clinical practice, patients with hyperuricemia or acute gout are advised to be monitored while using bempedoic acid (46).

From an etiological standpoint, the genesis of gout and hyperuricemia is attributable to various factors, notably including a definitive correlation with body mass. A research indicated that bariatric surgery markedly decreased body weight and serum urate concentrations in individuals suffering from obesity (47). The Mendelian randomization analysis further substantiates obesity as a contributory risk factor for the onset of gout and hyperuricemia (48). Multiple pathways may interfere in the mechanisms by which lipid-lowering drugs affect gout and urate. In a study of gout and urate and lipid profiles, gout and urate showed characteristic changes at different stages of the disease, and gout and hyperuricemia were associated with alterations in plasma lipid profiles, with reductions in LPC, LPC O- and LPCP- (LPC class as the mainpart ofoxidized LDL), underscoring the significance of monitoring LDL concentrations in individuals with gout and hyperuricemia. conversely, a significant association exists between gout and diabetes, as evidenced by studies demonstrating that individuals with diabetes exhibit a reduced risk of developing gout compared to non-diabetics (49, 50). Concurrently, lipid-lowering medications demonstrate both anti-inflammatory and pro-inflammatory properties (51), soluble urate and urate nanocrystals enhance NF-κB and IL-1β expression through NLRP3 inflammasome activation (52). PCSK9 promotes oxLDL-induced inflammation and TLR4 expression by increasing LOX expression, thereby initiating inflammation through NF-κB activation (53). This implication suggests that lipid-lowering drugs could modulate urate concentrations and gout symptoms via inflammatory routes, yet this hypothesis demands additional empirical substantiation. Concurrently, the dualistic character of statins, manifesting both anti-inflammatory and pro-inflammatory properties, intimates that inflammation might play a role in the genesis of gout, a hypothesis that requires further empirical validation (51).

This investigation showcased numerous notable strengths. Primarily, this research represents the inaugural systematic application of a drug-target Mendelian Randomization approach to elucidate the causal dynamics between lipid-lowering medications and both gout and urate. In the context of the prevailing absence of randomized controlled trials providing direct evidence, MR analysis serves to significantly diminish the impact of confounding variables typically present in observational studies by emulating a natural experiment akin to a randomized trial scenario, consequently enhancing the reliability of the conclusions.

Nevertheless, this study encompasses certain limitations. Firstly, the GWAS data utilized in this study predominantly originated from individuals of European descent. The limited diversity in current public GWAS databases restricts the availability of sufficient data from non-European populations, preventing this study from conducting an analysis across broader ethnic groups. Consequently, this limits the generalizability of our findings to other populations. Secondly, despite the application of both SMR and IVW-MR methods, only the IVW-MR presented a significant correlation, potentially due to the influence of various factors. Thirdly, our study primarily reflects the effects of lifetime suppression of drug targets on disease outcomes. Regarding long-term effects, the relationship between short-term drug use and disease risk remains uncertain. Mendelian Randomization studies may not fully capture the real-world effects of medication use due to factors such as dosage, mechanisms of action, individual variability, and duration of drug exposure. Consequently, there is a pressing need for high-quality randomized controlled trials to delve deeper into the specific impacts of lipid-lowering medications on gout and hyperuricemia, along with their underlying mechanisms. Fourthly, the absence of eQTL data for the target genes in the liver (a critical site for lipid metabolism) diminished the credibility of the observed correlations. Specifically, the limited sample size of NPC1L1 eQTL within the GTEx program, coupled with the absence of NPC1L1 eQTL data in blood samples, might have resulted in an underestimation of the efficacy of NPC1L1 inhibitors in managing gout and urate.

## 5 Conclusions

In conclusion, this research furnishes empirical evidence suggesting that HMGCR inhibitors elevate the risk of developing gout, while PCSK9 inhibitors heighten the risk of urate. Despite its limitations, this study offers significant insights into evaluating their application in personalized treatment strategies.

## Data Availability

The original contributions presented in the study are included in the article/**Supplementary Material**. Further inquiries can be directed to the corresponding author.
