# Supplementary material for: Associations between lipid-lowering drugs and urate and gout outcomes: a Mendelian randomization study
Source: Front Endocrinol (Lausanne). 2025 Jan 24;15:1398023. doi: 10.3389/fendo.2024.1398023 (PMC11802419; doi:10.3389/fendo.2024.1398023)
Supplement: Supplementary file 2 [file DataSheet1.docx]

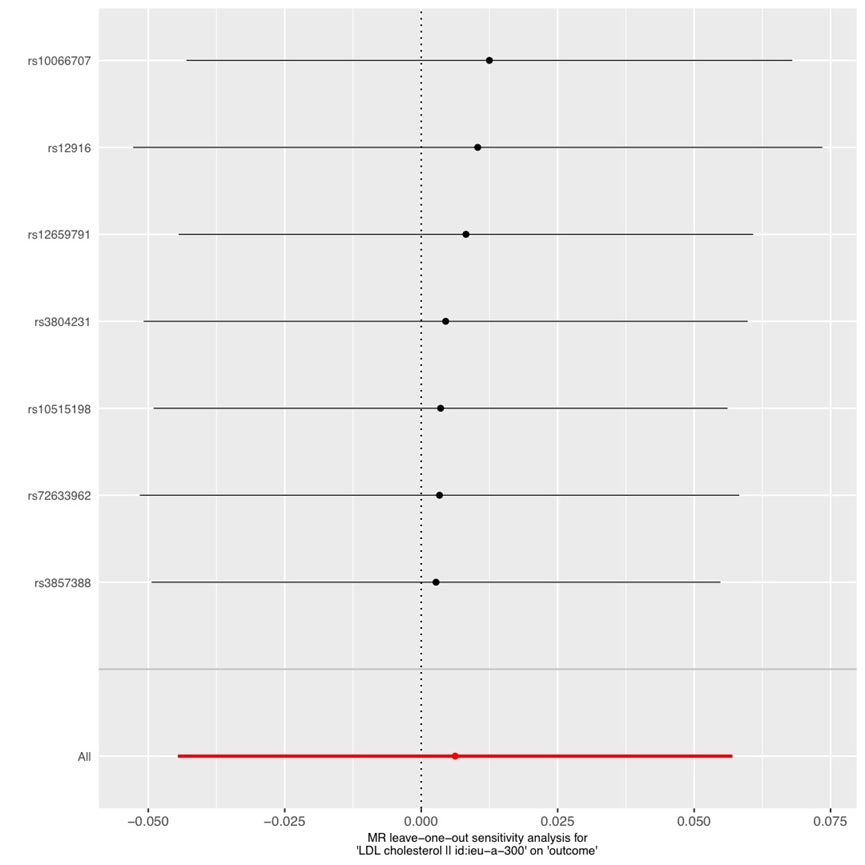


Supplementary Figure 1. Leave-one-out plot for sensitivity analysis of single SNP effect on “HMGCR”-to-“Urate” UVMR results.


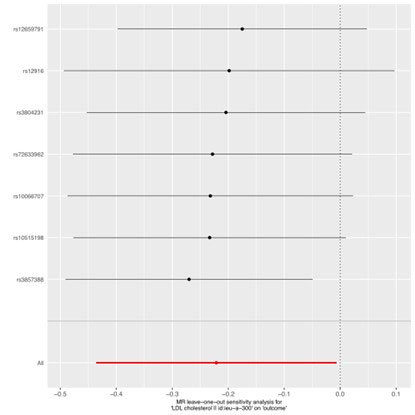


Supplementary Figure 2. Leave-one-out plot for sensitivity analysis of single SNP effect on “HMGCR”-to-“Gout” UVMR results.


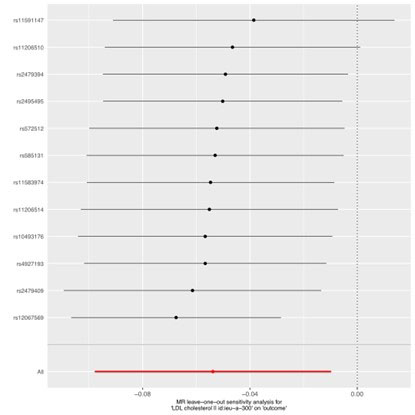


Supplementary Figure 3. Leave-one-out plot for sensitivity analysis of single SNP effect on “PCSK9”-to-“Urate” UVMR results.

**
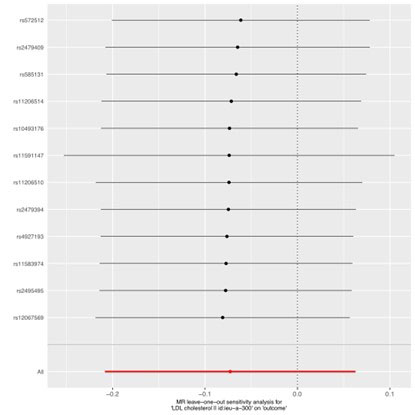
**

Supplementary Figure 4. Leave-one-out plot for sensitivity analysis of single SNP effect on “PCSK9”-to-“Gout” UVMR results.


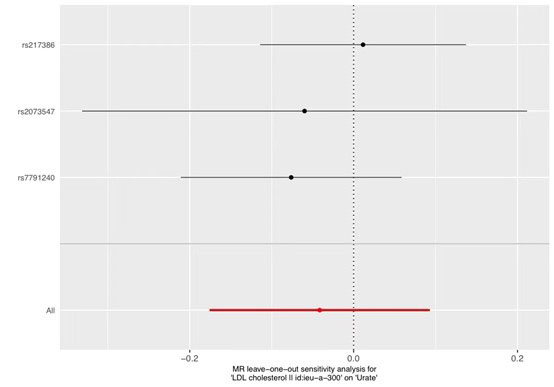


Supplementary Figure 5. Leave-one-out plot for sensitivity analysis of single SNP effect on “NPC1L1”-to-“Urate” UVMR results.


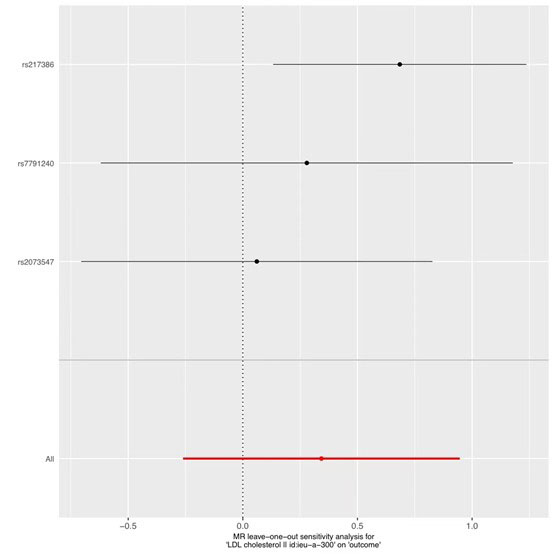


Supplementary Figure 6. Leave-one-out plot for sensitivity analysis of single SNP effect on “NPC1L1”-to-“Gout” UVMR results.
